# Hop and Acacia Phytochemicals Decreased Lipotoxicity in 3T3-L1 Adipocytes, *db/db* Mice, and Individuals with Metabolic Syndrome

**DOI:** 10.1155/2010/467316

**Published:** 2010-05-18

**Authors:** Deanna M. Minich, Robert H. Lerman, Gary Darland, John G. Babish, Linda M. Pacioretty, Jeffrey S. Bland, Matthew L. Tripp

**Affiliations:** ^1^Department of Research & Development, MetaProteomics, LLC, 9770 44th Avenue NW, Suite 100, Gig Harbor, WA 98332, USA; ^2^Bionexus, Cornell Technology Park, 30 Brown Road, Ithaca, NY 14850, USA

## Abstract

The plant-based compounds rho-iso-alpha acids (RIAA) from *Humulus lupulus* (hops) and proanthocyanidins (PAC) from *Acacia nilotica* have been shown to modulate insulin signaling in vitro. We investigated their effects on triglyceride (TG) deposition in 3T3-L1 adipocytes, glucose and insulin in obese mouse models, and metabolic syndrome markers in adults with metabolic syndrome. The combination of RIAA and PAC synergistically increased TG content and adiponectin secretion in 3T3-L1 adipocytes under hyperinsulinemic conditions and reduced glucose or insulin in obese mice. In a clinical trial, tablets containing 100 mg RIAA and 500 mg PAC or placebo were administered to metabolic syndrome subjects (3 tablets/day, *n* = 35; 6 tablets/day, *n* = 34; or placebo, *n* = 35) for 12 weeks. Compared to placebo, subjects taking 3 tablets daily showed greater reductions in TG, TG : HDL, fasting insulin, and HOMA scores. The combination of RIAA : PAC at 1 : 5 (wt : wt) favorably modulates dysregulated lipids in insulin resistance and metabolic syndrome.

## 1. Introduction

Metabolic syndrome, which affects almost 40% of American adults [1], is a complex metabolic mosaic of abnormal lipid, weight, and inflammatory markers. These metabolic abnormalities indicate underlying impairments in cellular insulin signaling and ultimately result in increased risk for diabetes or cardiovascular disease [2, 3]. Diet and exercise modification is recommended by American Heart Association as first-line treatment because of their ability to address one or more components. If lifestyle modification fails due to patient noncompliance, antidiabetic drugs are often prescribed. However, approximately half of patients require more than one pharmaceutical agent within three years of diagnosis, and the proportion increases to 75% within nine years [4]. 

The difficulty in treating metabolic syndrome and type 2 diabetes may be due to the failure to address underlying molecular mechanisms of insulin resistance, which remain not thoroughly understood. A relevant aspect of this pathology is that even before the development of fasting or postprandial hyperglycemia, insulin resistance manifests as abnormalities in triglyceride (TG) storage and lipolysis in insulin-sensitive tissues, causing disruption of insulin signaling, leading to activation of NF-*κ*B and the subsequent upregulation of proinflammatory genes [5, 6]. Targeting kinases related to insulin signaling and inflammation and/or reducing lipid overspill are potentially effective strategies to treat insulin resistance. 

Whereas kinase-inhibiting drugs may effectively completely inhibit these and other pathway networks, such strong inhibition has been associated with adverse events when used for a long duration. It has been shown that some botanical compounds derived from common foods have kinase-modulating activity or have insulin-potentiating action, safely and effectively modifying these interconnecting cellular pathways that address dysfunctional glucose metabolism, particularly as they relate to lipotoxicity [7–10]. In addition, they may also assist in peripheral mechanisms related to type 2 diabetes, such as improving lipid metabolism, antioxidant status, and vasculature [11, 12]. 

We previously discovered that traditionally consumed foodstuffs such as hops (*Humulus lupulus L.*) and acacia (*Acacia nilotica)* have modulating activity on kinases specific to insulin function. Specifically, hops-derived *rho*-iso-alpha acids (RIAAs), used as bitter flavoring agents in the beer industry, dose-dependently reduce GSK-3, PI3K, and PKC*β* activity in cell-free kinase assays [13]. Proanthocyanidin- (PAC-) rich extract from acacia bark was found to modulate the aforementioned kinases in addition to IKK*β* in a dose-dependent manner (unpublished). Others have also shown that PAC from a variety of botanicals improved symptoms of metabolic syndrome in vivo [14, 15]. 

In this paper, we report on our identification of a specific ratio of these natural products that favorably modified TG formation in the 3T3-L1 adipocyte model. Beneficial results with this ratio of actives on serum glucose and insulin in two diabetic mice models led us to conduct a 12-week clinical trial in individuals with the metabolic syndrome.

## 2. Materials and Methods

### 2.1. Chemicals and Reagents

Troglitazone, methylisobutylxanthine, dexamethasone, Oil Red O, and insulin were obtained from Sigma (St. Louis, MO). Penicillin, streptomycin, Dulbecco's modified Eagle's medium (DMEM) were from Mediatech (Herndon, VA) and 10% HI-FBS (heat inactivated fetal bovine serum) from Mediatech and Hyclone (Logan, UT). All standard reagents were obtained from Sigma and were of the highest purity commercially available. Hops RIAA and *Acacia* PAC were provided by Metagenics, Inc. (Gig Harbor, WA); their chemical structures have been previously described [16, 17]. Growth medium was made by adding 50 mL of HI-FBS and 5 mL of penicillin/streptomycin to 500 mL DMEM. This medium was stored at 4°C and warmed to 37°C in a water bath before use.

### 2.2. Cell Culture

The murine 3T3-L1 fibroblast cell line was purchased from American Type Culture Collection (Manassas, VA) and maintained according to instructions from the supplier. Preadipocytes were cultured in DMEM containing 10% HI-FBS, with added 50 U penicillin/mL and 50 *μ*g streptomycin/mL, and maintained in log phase prior to experimental setup. Cells were grown in a 5% CO_2_ humidified incubator at 37°C. 3T3-L1 cells were seeded at an initial density of ~4 ×10^4^ cells/cm^2^ in 24-well plates. For 2 days, the cells were allowed to grow to reach confluence. To force cells to differentiate into adipocytes, medium consisted of 10% FBS/DMEM (high glucose), 0.5 mM methylisobutylxanthine, 0.5 *μ*M dexamethasone, and 10 *μ*g/mL insulin was added. After 3 days, the medium was changed to postdifferentiation medium consisting of 10 *μ*g/mL insulin in 10% FBS/DMEM.

### 2.3. Lipogenic Activity in 3T3-L1 Cells

Lipogenesis was assessed using the preadipocyte differentiation assay as described by Xu et al. [18] with the following modification: (1) murine 3T3-L1 preadipocytes were selected rather than freshly isolated, rat preadipocytes, (2) intracellular lipid was measured using Oil Red O and BODIPY, and (3) troglitazone (10 *μ*M) was used as the positive control in place of rosiglitazone. Test material was added in dimethyl sulfoxide (DMSO) at day 0 of differentiation and every 2 days throughout the maturation phase (days 6/7). Fresh test material was added together with fresh media. Intracellular lipid was assessed using Oil Red O staining according to the method of Kasturi and Joshi [19]. Results were represented relative to stained cells in the solvent controls. 

### 2.4. Adiponectin Assay in 3T3-L1 Cells

For anti-inflammatory activity, on day 6 after differentiation, adipocytes were treated with test material 4 hours before addition of TNF-*α* at a final concentration of 10 ng/mL. Cells were incubated overnight for approximately 18 h, followed by removal of the supernatant medium and cell staining for nonpolar lipid with BODIPY. Adiponectin was quantified using the Quantikine Mouse IL-6 Immunoassay kit or the Mouse Adiponectin Quantikine Immunoassay kit (R&D Systems, Minneapolis, MN). 

### 2.5. Lipogenic Index, Adiponectin Index, and Synergy Calculations

For lipogenesis assays, test compounds were each assayed in duplicate for a minimum of three independent times. The Lipogenic Index was computed for each sample by normalizing Oil Red O values to the solvent control within each experiment. For adipogenic assays, each experiment was performed in duplicate. The effect of the test compounds on adiponectin secretion was computed relative to the solvent control. 

An estimate of the expected effects of the hops RIAA and acacia PAC combinations was made using the relationship 1/LI = *X*/LI_X_ + *Y*/LI_Y_ or 1/AI = *X*/AI_X_ + *Y*/AI_Y_ where LI = Lipogenic Index, AI = Adiponectin Index, and *X* and *Y* were relative fractions of each component in the test mixture and *X* + *Y* = 1. Synergy was inferred if the mean of the estimated LI and AI fell below the lower 95% confidence interval of the estimate of the corresponding observed fraction. This definition of synergy was described previously [20].

### 2.6. In Vivo Study

The effect of test materials on nonfasting serum glucose and insulin was assessed in the KK-A^y^/Ta and C57BLKS/J-m+/+ *Lep* *r* ^*db*^ (*db/db*) models of non-insulin-dependent diabetes mellitus (NIDDM) and obesity performed at MDS Pharma Services (Taiwan). Nine-week-old male KK-A^y^/Ta mice, weighing 40 ± 5 g, were provided by Clea Inc. (Tokyo, Japan); 9-week-old male *db/db *mice, weighing 50 ± 5 g, were provided by the Institute for Animal Reproduction (Ibaraki, Japan). These animals exhibited hyperinsulinemia, hyperglycemia, and islet atrophy. The animals were housed in Individually Ventilated Cages Racks (IVC Racks, 36 Mini Isolator systems) throughout the experiment. Each APEC cage (in cm, 26.7 length × 20.7 width × 14.0 height) was sterilized with autoclave and housed 5 mice. The mice were maintained in a hygienic environment under controlled temperature (22°–24°C) and humidity (60%–70%) with 12-hour light/dark cycle. The animals were fed sterilized lab chow and sterilized distilled water ad libitum. All aspects of this work, including housing, experimentation, and disposal of animals, were performed in accordance with the Guide for the Care and Use of Laboratory Animals (National Academy Press, Washington, D. C., 1996). 

Test substances and vehicle (2% Tween 80, Wako, Japan) were administered orally daily for 3 consecutive days starting immediately after the pretreatment blood sampling (day 1). Each substance was tested in a group of 5 mice. Post-treatment blood samples were drawn from the orbital sinus 90 minutes after administration of the final dose on day 7. Serum glucose and insulin levels were determined by enzymatic (Mutaratase-GOD, Wako, Japan) and ELISA (mouse insulin assay kit, SPIbio, France) methods, respectively. Post-treatment serum glucose and insulin values expressed in percentage of respective pretreatment values were calculated, and paired *t*-test was used for comparison. Differences were considered significant at *P* < .05 level (2-sided).

### 2.7. Human Clinical Trial

To investigate the effect of RIAA and PAC (supplemented orally at 1 : 5 wt : wt) on serum glucose, insulin, and lipids in individuals with metabolic syndrome, a randomized, 12-week, double-blind, placebo-controlled trial was conducted at the Functional Medicine Research Center (Gig Harbor, WA). Inclusion criteria included, (i) age 18–70 years, (ii) BMI 25–42.5 kg/m^2^ and (iii) TG : HDL-cholesterol ≥3.5; (iv) fasting insulin ≥71.75 pmol/L (or 10 *μ*IU/mL). In addition, individuals had to meet 3 of the following 5 criteria: (i) waist circumference >88.9 cm (or 35 inches) for women and >101.6 cm (or 40 inches) for men, (ii) TG ≥1.7 mmol/L (or 150 mg/dL), (iii) HDL-cholesterol <1.3 mmol/L (or 50 mg/dL) for women and <1.0 mmol/L (or 40 mg/dL for men, (iv) blood pressure ≥130/85 mm Hg or diagnosed hypertension on medication, and (v) fasting glucose ≥5.55 mmol/L (or 100 mg/dL). Key exclusion criteria included involvement in a weight loss program leading to 10% or greater body weight loss over the preceding 4 weeks, use of blood glucose or cholesterol-lowering medications or supplements, corticosteroid use in the preceding 4 weeks, allergy to study materials, or a history of diabetes, cancer, renal, hepatic, or cardiovascular diseases. The study was conducted in accordance with the Declaration of Helsinki, and informed written consent was obtained from each participant prior to enrollment in the study. 

Eligible participants were randomized to one of 4 arms using Microsoft Excel 2003 (Redmond, WA): Arm 1: placebo tablet, 3 times daily; Arm 2: active tablet consisting of hops 100 mg RIAA and 500 mg acacia PAC (1 : 5), 3 times daily; Arm 3: same as Arm 2 but at 2 tablets 3 times daily; Arm 4: placebo tablet, 2 tablets 3 times daily. All participants were instructed in an hour-long nutritional counseling session at the start of the study to follow the AHA Step 1 (low-fat) diet. Caloric prescriptions were determined by analyzing bioelectrical impedance to calculate basal metabolic rate (BMR) using the Katch-McArdle formula [21] BMR = 370 + (21.6 × lean mass in kg). The daily BMR was calculated by multiplying by an activity factor (1.12 for men and 1.14 for women) derived from the National Academy of Sciences low activity level (walking 2.2 miles daily) and was based on 150 minutes of aerobic activity per week. Caloric needs were estimated by subtracting 600 calories from the calculated BMR. 

After the baseline visit, participants returned at 2, 4, 8, and 12 weeks for follow-up visits. Compliance to the diet and exercise treatment was monitored at each visit using 3-day diet and exercise diaries. Overnight fasting blood samples were collected at baseline, 8 weeks and 12 weeks for lipid analysis. For 2-hour postprandial insulin/glucose response, participants consumed a solution containing 75 g glucose (Trutol 100, CASCO NERL Diagnostics) after the fasting blood draw, and 2 hours after the glucose challenge, blood was drawn and assayed for glucose and insulin levels (Laboratories Northwest, Tacoma, WA). 

### 2.8. Statistical Analysis

Data from Arm 1 and Arm 4 were combined and served as one placebo arm. For each variable, changes from baseline to 8 weeks and 12 weeks were calculated for each treatment arm. Baseline determinations were analyzed using one-way ANOVA. Changes from baseline to 8 weeks and 12 weeks were analyzed separately for each arm using a priori two-sided paired *t*-test. Differences among treatments were determined by ANOVA with post hoc multiple comparisons between treatments. All the tests were 2-sided. The *P*-value was significant if <.05. Data were analyzed using SAS (version 9.1, Cary, NC).

## 3. Results

### 3.1. RIAA and PAC Synergy In Vitro

The murine 3T3-L1 preadipocyte is used to study the potential effects of test compounds on adipocyte differentiation and adipogenesis. Assessing TG synthesis of 3T3-L1 cells provides a validated model of the insulin-sensitizing capacity of the test agent [22]. For assessing the effects of hops RIAA and acacia PAC combination on lipogenesis and adiponectin secretion, the 1-to-5 and 1-to-10 combinations of RIAA : PAC at 50, 10, 5, and 1 *μ*g/mL were tested. With respect to increasing lipid incorporation under the condition of hyperinsulinemia, RIAA : PAC at 1 : 5 exhibited synergistic effects at all 4 concentrations, while at 1 : 10 the synergy was observed at 5 and 10 *μ*g/mL (Table 1). Regarding increasing adiponectin secretion from TNF*α* stimulation, RIAA : PAC at 1 : 5 exhibited synergy at 1, 5, and 50 *μ*g/mL, while at 1 : 10 the synergy was observed at 1, 10, and 50 *μ*g/mL (Table 1).

### 3.2. RIAA and PAC Combinations on Serum Glucose and Insulin In Vivo

**Figure 1:**
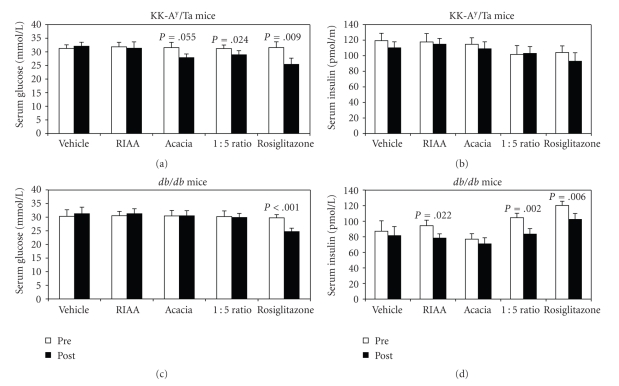
Nonfasting serum glucose and insulin concentration in KK-A^y^/Ta mice (a,b) and *db/db *mice (c,d).

With the RIAA : PAC at 1 : 5 and 1 : 10 showing synergistic effect on lipogenesis in vitro, we went on to test the effect of these ingredients (/) with both combined ratios as well as single ingredients on glucose metabolism in 2 NIDDM mouse models. Rosiglitazone was used as the positive control. In KK-A^y^/Ta mice, 3 days of RIAA or PAC treatment alone at 100 mg/kg did not reduce the glucose concentration, but RIAA : PAC at 1 : 5 significantly reduced glucose concentration from 31.20 ± 1.33 mmol/L to 28.92 ± 1.53 mmol/L (*P* = .024; Figure 1(a)). The combined ratio of 1 : 10 did not exhibit a significant effect (data now shown). The rosiglitazone treatment also significantly reduced glucose concentration. However, none of the treatments, including rosiglitazone, affected insulin concentrations in KK-A^y^/Ta mice (Figure 1(b)). In *db/db* mice, only rosiglitazone treatment significantly reduced glucose concentration (*P* < .001; Figure 1(c)). RIAA treatment at 100 mg/kg reduced insulin concentration from 94.2 ± 7.1 pmol/L to 78.4 ± 5.5 pmol/L (*P* = .022), while PAC treatment at 100 mg/kg did not have any effect. The RIAA : PAC at 1 : 5, however, significantly reduced insulin from 104.5 ± 5.7 pmol/L to 83.4 ± 7.0 pmol/L (*P* = .002), as did the rosiglitazone control (*P* = .006) (Figure 1(d)). The combined ratio of 1 : 10 did not significantly affect insulin and glucose concentrations (data not shown).

### 3.3. Human Clinical Trial

Given the observed synergistic effect of RIAA : PAC at 1 : 5 but not at 1 : 10 on glucose metabolism in 2 mouse models, we chose the 1 : 5 combination for our clinical trial. A total of 104 individuals were enrolled (35 for Arm 1 + 4, 35 for Arm 2, and 34 for Arm 3) and 91 completed the study (33 for Arm 1 + 4, 29 for Arm 2, and 29 for Arm 3). Baseline characteristics, fasting glucose and insulin and their 2-hour postprandial responses, and serum lipids did not differ by treatment arm (Table 2). As expected, participants exhibited high cholesterol, LDL, TG, and elevated blood pressure and were obese. There were no significant differences among arms at baseline in daily dietary caloric and nutrient intake except for soluble fiber (Table 2). 

Over time, total energy, carbohydrate, total fat, and saturated fat intake decreased in all arms, but the changes did not differ among arms (Table 3). Compared to baseline intake, Arms 1 + 4 (receiving placebo) had an increase in vegetable, soluble fiber, and insoluble fiber intake at 8 and/or 12 weeks, Arm 2 (receiving 3 active tablets daily) had an increase in vegetable intake at 8 weeks, and Arm 3 (receiving 6 active tablets daily) had an increase in soluble fiber and insoluble fiber intake at 8 and/or 12 weeks. Over time, individuals in all arms lost weight, waist circumference, and BMI at 8 weeks and 12 weeks compared to baseline although the degree of changes did not differ among arms. Some reductions in systolic and diastolic blood pressure were observed in Arms 1 + 4 and Arm 2 (Table 4). 

In terms of glucose metabolism (Table 4), Arms 1 and 4 participants showed an increase in fasting glucose at 8 weeks (*P* = .008) and 12 weeks (*P* = .013) compared to baseline; Arm 2 participants did not show any changes at 8 and 12 weeks; Arm 3 participants showed an increase at 8 weeks (*P* = .001) and 12 weeks (*P* = .014) compared to baseline. However, these values were within normal physiological ranges. The 2-hour post-prandial glucose values at 8 weeks and 12 weeks were similar to baseline in all 3 arms. Fasting insulin levels remained unchanged over time for Arm 1 + 4, but were significantly reduced at 8 weeks (*P* = .007) and 12 weeks (*P* = .045) for Arm 2. For Arm 3, the levels were also reduced at 8 weeks (*P* = .017) but not at 12 weeks (*P* = .81). The 2-hour post-prandial insulin values exhibited large variations but generally remained unchanged except being higher for Arm 1 + 4 at 12 weeks (*P* = .041). 

Of all the assessed lipid parameters (Table 3), serum TG exhibited the most significant differences over time. Compared to baseline, the TG level remained unchanged for Arm 1 + 4, decreased significantly at 8 weeks (by 0.55 ± 0.14 mmol/L; *P* < .001) and 12 weeks (by 0.59 ± 0.12 mmol/L; *P* < .001) for Arm 2, and decreased significantly at 8 weeks (by 0.50 ± 0.18 mmol/L; *P* = .011) but not 12 weeks (by 0.25 ± 0.24 mmol/L; *P* = .311) for Arm 3. Serum cholesterol was significantly reduced in Arm 1 + 4 at 12 weeks (*P* = .015) and moderately reduced in Arm 3 at 8 weeks (*P* = .065). Serum LDL remained unchanged in all 3 arms except for a trend toward reduction in Arm 1 + 4 at 12 weeks (*P* = .065). Serum HDL also remained unaffected in all 3 arms except for a trend toward reduction in Arm 3 at 8 weeks (*P* = .062). TG : HDL reflected the findings of lowered serum TG; a significant reduction was observed in Arm 2 at 8 and 12 weeks. A reduction in the HOMA scores was observed for Arm 2 at 8 weeks (*P* = .028).

## 4. Discussion

In the 3T3-L1 preadipocyte differentiation assay, a validated model of insulin-sensitizing capacity, the 1 : 5 combination of RIAA and PAC increased lipid incorporation under the condition of hyperinsulinemia in a synergistic manner at all four concentrations tested. This combination also demonstrated the ability to improve glucose metabolism in vivo in 2 type 2 diabetes mouse models: nonfasting serum glucose significantly decreased in KK-A^y^/Ta mice and non-fasting serum insulin significantly decreased in *db/db* mice after administration with RIAA : PAC at 1 : 5. Subjects with metabolic syndrome who took 100 mg RIAA and 500 mg PAC three times daily and followed the AHA Step 1 low-fat diet for 12 weeks experienced significant decreases in fasting TG and insulin levels compared to individuals who took placebo and followed the same diet. Subjects in this treatment group also showed greater reductions in TG : HDL and HOMA scores. Taken together, results of these three studies demonstrate the potential of this synergistic combination of phytonutrients to favorably modulate dysregulated lipids and insulin sensitivity in insulin resistance and metabolic syndrome. 

Mean TG levels in the United States have increased in recent decades, with elevated fasting TG (concentrations of 150 mg/dL or higher) now the norm in one out of three Americans [23]. Out of all the risk factors for metabolic syndrome, higher fasting TG, along with lower HDL and abdominal obesity, is the strongest predictor of metabolic syndrome development [24]. Other risk factors for metabolic syndrome, such as elevated blood pressure and altered glucose metabolism, differ more by age and sex and are not necessarily the strongest risk factors. In addition to predicting the risk of metabolic syndrome and new-onset diabetes [25], elevated fasting TG has been associated with 1.7 times increased risk for cardiovascular disease [26]. This increased risk is independent of LDL [27] and even HDL cholesterol levels [28]. Having both increased fasting TG levels and a high waist circumference are also independently associated with various other cardiometabolic risk factors, including small low-density lipoprotein particles, increased apolipoprotein B levels, increased insulin levels, reduced adiponectin concentrations [29], as well as subclinical vascular damage [30]. 

As research on the clinical and pathophysiological effects of elevated TG and systemic fatty acids on insulin resistance and metabolic syndrome continues, the traditional glucocentric perspective is being replaced. The processes of lipotoxicity and lipoapoptosis, in which the formation of reactive lipid particles promotes metabolically relevant cellular dysfunction and programmed cell death, are now known to be major mediators of insulin resistance, diabetes, and cardiovascular disease [31]. Unoxidized long-chain fatty acids overaccumulate, saturating the storage capacity of adipose tissue and leading to deleterious lipid spillover. The portal circulation becomes flooded with free fatty acids at metabolically inappropriate times when free fatty acids should be oxidized, thus exposing nonadipose tissues to fat excess. In the liver, muscle, heart, and pancreas, the excess lipids are driven into pathways which result in lipoapoptosis, ultimately leading to organ failure. The additional dysregulation of glucose homeostasis in combination with excess fatty acids provides an even greater synergistic effect leading to lipotoxicity and cell death. Therefore, TG and fatty acids should become a primary target for treating metabolic syndrome. 

Lifestyle modification with diet, regular exercise, and weight control, remains the primary intervention for lowering TG [23] and has proven effective in large prospective studies for prevention and treatment of the metabolic syndrome [32]. It is to be expected that a majority of persons will forgo long-term compliance with lifestyle changes alone, and it is probable that those taking pharmaceuticals will fail to adequately control the myriad metabolic imbalances manifest in the syndrome. Due to the high cost of metabolic syndrome, both in terms of human lives and monetary expenditures, it seems highly desirable to have safe, effective natural agents to support treatment. 

Animal and clinical studies with another hop compound, isohumulone, which is structurally similar to RIAA but less chemically stable [33], have revealed its ability to reduce levels of plasma TG, free fatty acids, plasma glucose, and HbA1c, improve glucose tolerance, and reduce insulin resistance [34, 35]. The in vitro and in vivo data from diabetic mouse models reported in the current study provides evidence for the hop compound RIAA for the insulin sensitizing capacity. 

Though we observed statistically significant responses in terms of TG, TG : HDL, and fasting insulin in subjects receiving RIAA (100 mg) : PAC (500 mg) three times daily at both 8 and 12 weeks, other variables, especially those related to glucose metabolism, did not improve in this arm and were actually less favorable in the arm receiving six tablets daily, as well as in the placebo arm. At least three explanations may account for this lack of favorable changes. First, subjects in all three arms had normal baseline glucose homeostasis—defined as a fasting plasma glucose level of less than 100 mg/dL and a 2-hour oral glucose tolerance test result of less than 140 mg/dL after a 75 g oral glucose load—with no evidence of impaired fasting glucose or impaired glucose tolerance. After treatment, fasting glucose remained within normal physiological ranges for the 3-tablets-daily and placebo arms and increased only slightly above normal in the 6-tablets-daily arm despite the statistically significant increases from baseline in the placebo and 6-tablets-daily-arms. Because fasting glucose and glucose tolerance started and remained primarily within normal physiological ranges, and because changes were so slight, they may be considered clinically insignificant. Second, it has been shown that metabolic parameters such as fasting glucose are not the primary predictors of the metabolic syndrome [24] and that insulin sensitivity can already be substantially decreased within the normal range of fasting and 2-hour glucose [36]. As the roles of free fatty acids and insulin resistance in cardiometabolic risk continue to be refined, it is becoming apparent that treating elevated TG and associated lipotoxicity is important, independent of glucose metabolism. In the 3-tablets daily arm, the combination of normal glucose metabolism with significant improvements in TG and fasting insulin indicates the possibility that patients were mildly insulin sensitive but had not yet developed overt problems with glucose homeostasis. Perhaps extending RIAA/PAC treatment duration beyond 12 weeks or studying patients with more advanced glucose homeostasis dysregulation at baseline would have achieved more significant results in terms of measures of both glucose and lipid metabolism. Third, although the subjects' compliance with dietary and exercise recommendations was satisfactory, it is possible that the AHA Step 1 (low-fat) diet, even when combined with these supplemental phytonutrients, was inadequate for producing greater changes in parameters related to glucose, insulin, or lipids. The AHA Step 1 diet, primarily intended as the starting point for treating hypercholesterolemia, restricts total fat to no more than 30 percent of total calories, saturated fat to no more than 10 percent of total calories, and cholesterol to less than 300 mg/day [37]. It has been found to lower both serum total cholesterol and LDL cholesterol [38] but has also been associated with lowering serum HDL cholesterol [39] as well as with nutritional inadequacies [40] and has since been replaced by new, more comprehensive AHA dietary recommendations as part of Therapeutic Lifestyle Changes [41]. More comprehensive diets, such as those which reduce glycemic load and increase fiber, may be required in combination with supplemental phytonutrients such as RIAA : PAC in order to achieve more substantial reductions in established risk markers for cardiovascular disease and type 2 diabetes. 

The favorable results for TG and fasting insulin observed in the arm receiving RIAA : PAC three times daily did not carry over to those receiving the tablets six times daily. The plateau may have simply been reached using the lower dosage method although this does not explain the fact that we did not observe even similar benefits in the higher-dose arm. Alternate explanations include the possibility of an absorption issue at this higher dose, or, more likely, could be related to the slightly higher serum TG and fasting insulin levels observed in the 3-tablets-daily arm at baseline. These mildly higher values may be more likely to respond to this phytonutrient combination, regardless of the dose. Both pharmacokinetic studies as well as larger controlled studies in patients with more advanced hypertriglyceridemia and glucose dysregulation at baseline would provide further insight into the dosage issue. The current data indicates that three tablets daily provide maximum physiological impact, and higher oral doses do not increase clinical benefit.

## 5. Conclusions

The continuing epidemic of metabolic syndrome, which substantially increases the risk of type II diabetes and cardiovascular disease, calls for novel, effective therapeutic approaches beyond pharmaceuticals and lifestyle modification. The addition of certain phytochemicals such as RIAA from hops and PAC from acacia to dietary and lifestyle modification has the potential to more favorably modulate insulin signaling and to decrease the deleterious effects of lipotoxicity which characterize metabolic syndrome. We showed that the specific combination of RIAA : PAC (1 : 5) synergistically increased TG content in 3T3-L1 adipocytes under conditions of hyperinsulinemia and increased adiponectin secretion in cells treated with TNF-*α*. Serum glucose or insulin concentrations in KK-A^y^/Ta and *db/db* mice were reduced by three-day oral treatment with such combination at 100 mg/kg body weight more than by treatments with individual compounds. Daily supplementation with 300 mg RIAA and 1500 mg PAC in addition to lifestyle modification including dietary alteration reduces serum TG, TG : HDL, and fasting insulin significantly more than diet and lifestyle modification alone in patients with features of the metabolic syndrome. This phytonutrient combination provides a potential therapy for correcting or modulating dysregulated lipids and improving insulin sensitivity in metabolic syndrome.

## Figures and Tables

**Table 1:** Observed and expected lipogenic response elicited by hops RIAA and acacia PAC in the 3T3-L1 model.

	RIAA : PAC at 1 : 5 (wt : wt)			RIAA : PAC at 1 : 10 (wt : wt)		
Lipogenic Index*	Observed	Expected	Synergy	Observed	Expected	Synergy
50 (*μ*g/mL)	1.05	0.98	Yes	0.99	1.03	No
10	0.96	0.89	Yes	1.00	0.90	Yes
5	0.93	0.90	Yes	1.00	0.90	Yes
1	0.92	0.89	Yes	0.94	0.89	No

Adiponectin Index**						
50 (*μ*g/mL)	1.27	1.08	Yes	1.29	1.11	Yes
10	0.99	1.25	No	1.07	0.95	Yes
5	1.02	0.92	Yes	0.94	1.06	No
1	1.19	1.07	Yes	1.03	0.94	Yes

*Lipogenic Index = [optical density]_test_/[optical density]_DMSO control_.

**Adipogenic Index = [adiponectin]_test_/[adiponectin]_TNF*α* control._

**Table 2:** Baseline anthropometrics, dietary intake, glucose metabolism, and lipid parameters of study participants by treatment arm.

	Arm 1 + 4 (placebo)	Arm 2 (3 tablets)	Arm 3 (6 tablets)	*P*-Value
Sex				
Men	11	12	11	
Women	24	23	23	

Age (y)	46.0 ± 2.2	47.9 ± 2.3	45.1 ± 2.0	.65
Weight (kg)	100.1 ± 2.7	99.6 ± 2.4	100.9 ± 3.0	.95
Waist (cm)	108.9 ± 2.1	108.9 ± 1.9	109.2 ± 2.2	.99
BMI (kg/m^2^)	35.0 ± 0.7	35.4 ± 0.7	35.4 ± 0.8	.89

Total energy (kJ)	8303 ± 460	8948 ± 427	8224 ± 670	.45
Carbohydrate (g)	232.6 ± 13.6	257.6 ± 18.8	257.8 ± 24.6	.56
Protein (g)	81.5 ± 4.7	87.0 ± 4.4	85.5 ± 5.3	.70
Total fat (g)	81.3 ± 5.8	83.1 ± 5.1	93.4 ± 7.4	.33
Saturated fat (g)	27.5 ± 2.1	27.5 ± 2.1	31.7 ± 2.7	.33
Fruit (serving)	2.3 ± 0.3	2.5 ± 0.3	2.8 ± 0.4	.53
Vegetable (serving)	3.1 ± 0.3	3.4 ± 0.3	3.2 ± 0.2	.67
Soluble fiber (g)	1.7 ± 0.2	2.7 ± 0.6	1.4 ± 0.1	.03
Insoluble fiber (g)	4.4 ± 0.4	5.0 ± 0.7	4.2 ± 0.4	.54

Blood pressure (mmHg)				
Systolic	131.0 ± 2.6	129.7 ± 2.4	127.5 ± 3.0	.64
Diastolic	83.7 ± 1.4	82.6 ± 1.3	83.7 ± 1.8	.83
F. glucose (mmol/L)	5.35 ± 0.09	5.50 ± 0.10	5.38 ± 0.09	.53
Glucose 2pp (mmol/L)	6.06 ± 0.29	7.13 ± 0.37	6.77 ± 0.44	.11
F. insulin (pmol/L)	94.7 ± 6.5	125.3 ± 15.9	99.2 ± 9.9	.12
Insulin 2pp (pmol/L)	575.5 ± 65.1	820.9 ± 132.4	599.4 ± 81.8	.14
HbA1c, %	5.61 ± 0.06	5.68 ± 0.07	5.70 ± 0.07	.63
Cholesterol (mmol/L)	5.48 ± 0.18	5.41 ± 0.17	5.46 ± 0.17	.96
LDL (mmol/L)	3.20 ± 0.14	3.10 ± 0.15	3.19 ± 0.16	.88
TG (mmol/L)	2.61 ± 0.26	2.89 ± 0.25	2.52 ± 0.18	.53
HDL (mmol/L)	1.07 ± 0.03	1.07 ± 0.03	1.09 ± 0.03	.83
Chol : HDL	5.2 ± 0.2	5.1 ± 0.2	5.0 ± 0.2	.74
TG : HDL	5.8 ± 0.6	6.3 ± 0.6	5.4 ± 0.4	.46

**Table 3:** Changes in dietary intake at 8 weeks and 12 weeks by treatment arm.

	Arm 1 + 4 (placebo)		Arm 2 (3 tablets)		Arm 3 (6 tablets)		*P*-value	*P*-value	*P*-value
	Value	Change from baseline	Value	Change from baseline	Value	Change from baseline	(1 + 4 versus 2)	(1 + 4 versus 3)	(2 versus 3)
Total energy (kJ)									
8 weeks	6365 ± 230	−**1946 ± 427** ^†^	6231 ± 347	−2**791 ± 456** ^†^	6215 ± 331	−**3139 ± 716** ^†^	.66	.27	.12
12 weeks	6169 ± 306	−**2310 ± 519** ^†^	6353 ± 310	−**2745 ± 452** ^†^	6378 ± 431	−**2976 ± 716** ^†^	.78	.46	.30
Carbohydrate (g)									
8 weeks	198.0 ± 9.1	−32.6 ± 13.9*	190.7 ± 12.3	−73.8 ± 19.5^†^	183.8 ± 10.5	−79.8 ± 25.7^†^	.83	.14	.09
12 weeks	192.8 ± 10.4	−37.7 ± 16.2*	191.2 ± 10.6	−**76.0 ± 18.1** ^†^	193.9 ± 13.9	−69.7 ± 26.2*	.83	.18	.26
Protein (g)									
8 weeks	78.8 ± 3.4	−3.8 ± 4.9	77.3 ± 3.2	−10.1 ± 4.0*	79.0 ± 4.1	−5.3 ± 5.5	.50	.36	.83
12 weeks	74.6 ± 3.9	−8.0 ± 5.8	75.9 ± 4.2	−11.9 ± 4.4*	81.5 ± 4.8	−2.8 ± 6.3	.27	.62	.51
Total fat (g)									
8 weeks	48.1 ± 2.4	−**33.6 ± 5.6** ^†^	48.7 ± 3.9	−**33.6 ± 6.6** ^†^	50.4 ± 3.6	−**44.5 ± 8.5** ^†^	.28	.99	.27
12 weeks	47.3 ± 3.5	−**34.4 ± 6.0** ^†^	51.1 ± 3.3	−**32.0 ± 6.1** ^†^	50.0 ± 4.6	−**44.9 ± 7.7** ^†^	.18	.80	.26
Saturated fat (g)									
8 weeks	16.0 ± 0.9	−**11.9 ± 2.1** ^†^	14.9 ± 1.4	−**12.7 ± 2.7** ^†^	15.3 ± 1.2	−**17.1 ± 3.2** ^†^	.25	.85	.16
12 weeks	16.7 ± 1.4	−**11.3 ± 2.3** ^†^	15.2 ± 1.2	−**12.5 ± 2.6** ^†^	15.3 ± 1.5	−**17.1 ± 2.8** ^†^	.21	.74	.11
Fruit (serving)									
8 weeks	2.6 ± 0.3	0.02 ± 0.33	2.6 ± 0.3	−0.03 ± 0.48	2.2 ± 0.2	−0.1 ± 0.3	.87	.93	.80
12 weeks	2.3 ± 0.2	−0.04 ± 0.34	2.3 ± 0.3	−0.30 ± 0.44	2.1 ± 0.3	−0.3 ± 0.4	.96	.63	.68
Vegetable (serving)									
8 weeks	4.2 ± 0.3	**1.1 ± 0.4** ^†^	4.3 ± 0.4	1.0 ± 0.4*	3.5 ± 0.3	0.3 ± 0.3	.18	.74	.09
12 weeks	3.5 ± 0.4	0.4 ± 0.4	4.2 ± 0.3	0.8 ± 0.4	3.9 ± 0.3	0.7 ± 0.4	.93	.57	.63
Soluble fiber (g)									
8 weeks	2.7 ± 0.3	**0.9 ± 0.3** ^†^	2.4 ± 0.2	−0.7 ± 0.7	2.0 ± 0.2	**0.6 ± 0.2** ^†^	**.04**	**.01**	.65
12 weeks	2.7 ± 0.2	**0.9 ± 0.3** ^†^	2.5 ± 0.2	−0.6 ± 0.6	2.7 ± 0.3	**1.4 ± 0.3** ^†^	<**.01**	**.01**	.42
Insoluble fiber (g)									
8 weeks	7.1 ± 0.6	**2.6 ± 0.7** ^†^	5.8 ± 0.4	0.4 ± 0.8	5.3 ± 0.6	1.2 ± 0.6	.44	**.03**	.14
12 weeks	6.4 ± 0.6	1.9 ± 0.8*	6.1 ± 0.5	0.7 ± 0.8	6.5 ± 0.6	**2.4 ± 0.6** ^†^	.11	.21	.68

Data expressed as mean ± SE. *P < .05, ^†^ *P* < .01 compared to baseline.

**Table 4:** Changes in anthropometric, glucose metabolism, and lipid variables at 8 and 12 weeks by treatment arm.

	Arm 1 + 4 (placebo)		Arm 2 (3 tablets)		Arm 3 (6 tablets)		*P*-value	*P*-value	*P*-value
	Value	Change from baseline	Value	Change from baseline	Value	Change from baseline	(1+4 versus 2)	(1+4 versus 3)	(2 versus 3)
Weight (kg)									
8 weeks	96.6 ± 2.8	−**3.5 ± 0.5** ^†^	94.9 ± 2.4	−**3.1 ± 0.5** ^†^	100.2 ± 3.1	−**3.3 ± 0.5** ^†^	.50	.77	.71
12 weeks	96.2 ± 2.8	−**3.9 ± 0.6** ^†^	95.9 ± 2.4	−**3.2 ± 0.5** ^†^	98.9 ± 3.1	−**3.8 ± 0.6** ^†^	.39	.86	.50
Waist (cm)									
8 weeks	105.9 ± 2.2	−**3.4 ± 0.7** ^†^	104.6 ± 2.0	−**2.6 ± 0.7** ^†^	107.7 ± 2.3	−**2.4 ± 0.7** ^†^	.84	.43	.33
12 weeks	105.4 ± 2.2	−**3.9 ± 0.7** ^†^	105.1 ± 2.1	−**3.4 ± 0.8** ^†^	107.0 ± 2.2	−**2.8 ± 0.7** ^†^	.56	.64	.29
BMI (kg/m^2^)									
8 weeks	33.7 ± 0.7	−**1.2 ± 0.2** ^†^	33.7 ± 0.7	−**1.1 ± 0.2** ^†^	34.6 ± 0.8	−**1.2 ± 0.2** ^†^	.60	.77	.83
12 weeks	33.6 ± 0.8	−**1.4 ± 0.2** ^†^	34.1 ± 0.7	−**1.1 ± 0.2** ^†^	34.2 ± 0.8	−**1.3 ± 0.2** ^†^	.43	.83	.58
BP-systolic (mmHg)									
8 weeks	126.9 ± 2.5	−3.4 ± 1.3*	124.7 ± 2.7	−**4.7 ± 1.5** ^†^	127.1 ± 2.5	−2.0 ± 2.3	.59	.57	.29
12 weeks	127.9 ± 2.4	−2.3 ± 1.4	127.9 ± 2.4	−0.6 ± 1.9	128.1 ± 2.3	−0.1 ± 3.1	.58	.47	.87
BP-diastolic (mmHg)									
8 weeks	79.1 ± 1.4	−**4.9 ± 1.3** ^†^	80.3 ± 1.5	−2.5 ± 1.0*	82.8 ± 1.5	−2.3 ± 1.2	.17	.13	.88
12 weeks	80.6 ± 1.6	−3.4 ± 1.3*	81.3 ± 1.6	−2.0 ± 1.2	81.0 ± 2.0	−3.3 ± 1.9	.51	.98	.54
F. glucose (mmol/L)									
8 weeks	5.52 ± 0.09	**0.16 ± 0.06** ^†^	5.55 ± 0.13	0.07 ± 0.09	5.64 ± 0.07	**0.29 ± 0.08** ^†^	.40	.23	**.05**
12 weeks	5.54 ± 0.09	0.18 ± 0.07*	5.52 ± 0.10	0.03 ± 0.08	5.62 ± 0.13	0.24 ± 0.09*	.19	.59	.08
Glucose 2pp (mmol/L)									
8 weeks	6.49 ± 0.29	0.43 ± 0.29	6.97 ± 0.36	−0.13 ± 0.35	7.21 ± 0.41	0.47 ± 0.33	.22	.94	.21
12 weeks	6.57 ± 0.33	0.51 ± 0.34	6.81 ± 0.27	−0.36 ± 0.32	6.81 ± 0.44	0.04 ± 0.34	.07	.32	.41
F. insulin (pmol/L)									
8 weeks	86.3 ± 10.5	−8.4 ± 6.7	93.8 ± 11.1	−**27.2 ± 9.4** ^†^	79.6 ± 5.4	−20.6 ± 8.1*	.10	.28	.57
12 weeks	87.0 ± 8.5	−7.7 ± 6.5	107.0 ± 14.2	−20.5 ± 9.8*	96.4 ± 16.6	−2.8 ± 11.3	.32	.71	.19
Insulin 2pp (pmol/L)									
8 weeks	460.8 ± 77.5	−114.7 ± 63.5	579.7 ± 89.1	−213.9 ± 118.9	550.4 ± 101.7	−57.5 ± 63.9	.40	.63	.21
12 weeks	705.8 ± 85.0	130.3 ± 61.2*	863.0 ± 117.6	35.4 ± 72.6	655.9 ± 82.5	56.5 ± 61.0	.30	.42	.82
Cholesterol (mmol/L)									
8 weeks	5.35 ± 0.20	−0.13 ± 0.13	5.26 ± 0.19	−0.19 ± 0.13	5.20 ± 0.24	−0.26 ± 0.14	.73	.47	.72
12 weeks	5.16 ± 0.17	−0.32 ± 0.12*	5.17 ± 0.16	−0.18 ± 0.11	5.30 ± 0.23	−0.16 ± 0.13	.42	.35	.90
LDL (mmol/L)									
8 weeks	3.24 ± 0.16	−0.08 ± 0.12	3.15 ± 0.18	0.06 ± 0.12	3.23 ± 0.20	−0.10 ± 0.11	.40	.91	.35
12 weeks	2.98 ± 0.14	−0.24 ± 0.12	3.08 ± 0.15	0.10 ± 0.11	3.24 ± 0.17	0.02 ± 0.11	**.04**	.10	.64
TG (mmol/L)									
8 weeks	2.60 ± 0.36	−0.02 ± 0.39	2.37 ± 0.21	−**0.55 ± 0.14** ^†^	2.05 ± 0.19	−0.50 ± 0.18*	.17	.22	.89
12 weeks	2.41 ± 0.23	−0.20 ± 0.19	2.28 ± 0.22	−0.59 ± 0.12^†^	2.27 ± 0.32	−0.25 ± 0.24	.14	.87	.21
HDL (mmol/L)									
8 weeks	1.07 ± 0.04	0.01 ± 0.02	1.06 ± 0.03	−0.01 ± 0.02	1.05 ± 0.03	−0.04 ± 0.02	.56	.13	.36
12 weeks	1.07 ± 0.04	0.00 ± 0.02	1.07 ± 0.03	−0.00 ± 0.02	1.06 ± 0.04	−0.03 ± 0.03	.83	.29	.40
Chol/HDL									
8 weeks	5.1 ± 0.2	−0.2 ± 0.1	5.0 ± 0.2	−0.1 ± 0.1	5.0 ± 0.2	−0.1 ± 0.1	.90	.59	.69
12 weeks	4.9 ± 0.2	−0.3 ± 0.1*	4.9 ± 0.2	−0.2 ± 0.1	5.1 ± 0.2	0.0 ± 0.1	.41	.06	.30
TG/HDL									
8 weeks	5.9 ± 1.0	0.1 ± 1.1	5.3 ± 0.6	−**1.1 ± 0.4** ^†^	4.6 ± 0.4	−0.8 ± 0.4	.24	.37	.79
12 weeks	5.3 ± 0.5	−0.5 ± 0.5	5.0 ± 0.5	−**1.3 ± 0.3** ^†^	5.1 ± 0.8	−0.3 ± 0.6	.23	.76	.15
HOMA									
8 weeks	3.0 ± 0.4	−0.2 ± 0.3	3.3 ± 0.4	−0.9 ± 0.4*	2.8 ± 0.2	−0.6 ± 0.3	.12	.37	.51
12 weeks	3.1 ± 0.3	−0.1 ± 0.3	3.7 ± 0.5	−0.7 ± 0.4	3.7 ± 0.9	0.3 ± 0.6	.38	.49	.13

Data expressed as mean ± SE. *P < .05, ^†^ *P* < .01 compared to baseline.

## References

[1] Ford ES (2005). Prevalence of the metabolic syndrome defined by the international diabetes federation among adults in the U.S.. *Diabetes Care*.

[2] Alexander CM, Landsman PB, Teutsch SM, Haffner SM (2003). NCEP-defined metabolic syndrome, diabetes, and prevalence of coronary heart disease among NHANES III participants age 50 years and older. *Diabetes*.

[3] Isomaa B, Almgren P, Tuomi T (2001). Cardiovascular morbidity and mortality associated with the metabolic syndrome. *Diabetes Care*.

[4] Turner RC, Cull CA, Frighi V, Holman RR (1999). Glycemic control with diet, sulfonylurea, metformin, or insulin in patients with type 2 diabetes mellitus. Progressive requirement for multiple therapies (UKPDS 49). *Journal of the American Medical Association*.

[5] McGarry JD (2002). Dysregulation of fatty acid metabolism in the etiology of type 2 diabetes. *Diabetes*.

[6] Schinner S, Scherbaum WA, Bornstein SR, Barthel A (2005). Molecular mechanisms of insulin resistance. *Diabetic Medicine*.

[7] Ivorra MD, Paya M, Villar A (1989). A review of natural products and plants as potential antidiabetic drugs. *Journal of Ethnopharmacology*.

[8] Jia W, Gaoz W, Tang L (2003). Antidiabetic herbal drugs officially approved in China. *Phytotherapy Research*.

[9] Jung M, Park M, Lee HC, Kan Y-H, Kang ES, Kim SK (2006). Antidiabetic agents from medicinal plants. *Current Medicinal Chemistry*.

[10] Wang HX, Ng TB (1999). Natural products with hypoglycemic, hypotensive, hypocholesterolemic, antiatherosclerotic and antithrombotic activities. *Life Sciences*.

[11] Anderson DC (2005). Pharmacologic prevention or delay of type 2 diabetes mellitus. *The Annals of Pharmacotherapy*.

[12] Swanston-Flatt SK, Day C, Flatt PR, Gould BJ, Bailey CJ (1989). Glycaemic effects of traditional European plant treatments for diabetes. Studies in normal and streptozotocin diabetic mice. *Diabetes Research*.

[13] Tripp ML, Konda VR, Darland G (2009). Rho-iso-*α* acids and tetrahydro-iso-*α* acids are selective protein kinase inhibitors which potently reduce inflammation in macrophages in vitro and in the collagen-induced rheumatoid arthritis model in vivo. *Acta Hort (ISHS)*.

[14] Preuss HG, Bagchi D, Bagchi M (2002). Protective effects of a novel niacin-bound chromium complex and a grape seed proanthocyanidin extract on advancing age and various aspects of syndrome X. *Annals of the New York Academy of Sciences*.

[15] Tsai H-Y, Wu L-Y, Hwang LS (2008). Effect of a proanthocyanidin-rich extract from longan flower on markers of metabolic syndrome in fructose-fed rats. *Journal of Agricultural and Food Chemistry*.

[16] Hall AJ, Babish JG, Darland GK (2008). Safety, efficacy and anti-inflammatory activity of rho iso-*α*-acids from hops. *Phytochemistry*.

[17] Lerman RH, Minich DM, Darland G (2008). Enhancement of a modified mediterranean-style, low glycemic load diet with specific phytochemicals improves cardiometabolic risk factors in subjects with metabolic syndrome and hypercholesterolemia in a randomized trial. *Nutrition & Metabolism*.

[18] Xu M-E, Xiao S-Z, Sun Y-H, Ou-Yang Y, Guan C, Zheng X-X (2006). A preadipocyte differentiation assay as a method for screening potential anti-type II diabetes drugs from herbal extracts. *Planta Medica*.

[19] Kasturi R, Joshi VC (1982). Hormonal regulation of stearoyl coenzyme a desaturase activity and lipogenesis during adipose conversion of 3T3-L1 cells. *Journal of Biological Chemistry*.

[20] Berenbaum MC (1989). What is synergy?. *Pharmacological Reviews*.

[21] Katch FI, McArdle WD (1975). Validity of body composition prediction equations for college men and women. *American Journal of Clinical Nutrition*.

[22] Raz I, Eldor R, Cernea S, Shafrir E (2005). Diabetes: insulin resistance and derangements in lipid metabolism. Cure through intervention in fat transport and storage. *Diabetes/Metabolism Research and Reviews*.

[23] Ford ES, Li C, Zhao G, Pearson WS, Mokdad AH (2009). Hypertrigly ceridemia and its pharmacologic treatment among US adults. *Archives of Internal Medicine*.

[24] Scuteri A, Morrell CH, Najjar SS (2009). Longitudinal paths to the metabolic syndrome: can the incidence of the metabolic syndrome be predicted? The Baltimore longitudinal study of aging. *Journals of Gerontology*.

[25] Sattar N, McConnachie A, Shaper AG (2008). Can metabolic syndrome usefully predict cardiovascular disease and diabetes? Outcome data from two prospective studies. *Lancet*.

[26] Stalenhoef AFH, de Graaf J (2008). Association of fasting and nonfasting serum triglycerides with cardiovascular disease and the role of remnant-like lipoproteins and small dense LDL. *Current Opinion in Lipidology*.

[27] Kannel WB, Vasan RS (2009). Triglycerides as vascular risk factors: new epidemiologic insights. *Current Opinion in Cardiology*.

[28] Hokanson JE, Austin MA (1996). Plasma triglyceride level is a risk factor for cardiovascular disease independent of high-density lipoprotein cholesterol level: a meta-analysis of population-based prospective studies. *Journal of Cardiovascular Risk*.

[29] Blackburn P, Lemieux I, Almeras N (2009). The hypertriglyceridemic waist phenotype versus the National Cholesterol Education Program-Adult Treatment Panel III and International Diabetes Federation clinical criteria to identify high-risk men with an altered cardiometabolic risk profile. *Metabolismml: Clinical and Experimental*.

[30] Fantin F, Di Francesco V, Rossi A (2010). Abdominal obesity and subclinical vascular damage in the elderly. *Journal of Hypertension*.

[31] Kusminski CM, Shetty S, Orci L, Unger RH, Scherer PE (2009). Diabetes and apoptosis: lipotoxicity. *Apoptosis*.

[32] Bo S, Ciccone G, Baldi C (2007). Effectiveness of a lifestyle intervention on metabolic syndrome. A randomized controlled trial. *Journal of General Internal Medicine*.

[33] De Keukeleirc D (2000). Fundamentals of beer and hop chemistry. *Química Nova*.

[34] Shimura M, Hasumi A, Minato T (2005). Isohumulones modulate blood lipid status through the activation of PPAR*α*. *Biochimica et Biophysica Acta*.

[35] Yajima H, Ikeshima E, Shiraki M (2004). Isohumulones, bitter acids derived from hops, activate both peroxisome proliferator-activated receptor *α* and *γ* and reduce insulin resistance. *Journal of Biological Chemistry*.

[36] Stancakova A, Javorsky M, Kuulasmaa T, Haffner SM, Kuusisto J, Laakso M (2009). Changes in insulin sensitivity and insulin release in relation to glycemia and glucose tolerance in 6,414 finnish men. *Diabetes*.

[38] Geil PB, Anderson JW, Gustafson NJ (1995). Women and men with hypercholesterolemia respond similarly to an American Heart Association step 1 diet. *Journal of the American Dietetic Association*.

[39] Bunyard LB, Dennis KE, Nicklas BJ (2002). Dietary intake and changes in lipoprotein lipids in obese, postmenopausal women placed on an American Heart Association Step 1 diet. *Journal of the American Dietetic Association*.

[40] Bae CY, Keenan JM, Fontaine P, Wenz J, Ripsin CM, McCaffrey DJ (1993). Plasma lipid response and nutritional adequacy in hypercholesterolemic subjects on the American Heart Association Step-One Diet. *Archives of Family Medicine*.

[41] Lichtenstein AH, Appel LJ, Brands M (2006). Diet and lifestyle recommendations revision 2006: a scientific statement from the American Heart Association Nutrition Committee. *Circulation*.

